# Murine mammary stem/progenitor cell isolation: Different method matters?

**DOI:** 10.1186/s40064-016-1787-3

**Published:** 2016-02-20

**Authors:** Hui Gao, Qiaoxiang Dong, Yuanhong Chen, Fuchuang Zhang, Anqi Wu, Yuanshuo Shi, Abhik Bandyopadhyay, Benjamin J. Daniel, Changjiang Huang, Lu-Zhe Sun

**Affiliations:** School of Laboratory Medicine and Life Science, Wenzhou Medical University, University Town, Wenzhou, 325035 China; Department of Cellular and Structural Biology, University of Texas Health Science Center, San Antonio, TX 78299 USA; Institute of Environmental Safety and Human Health, Wenzhou Medical University, University Town, Wenzhou, 325035 China; Flow Cytometry Facility, University of Texas Health Science Center, San Antonio, TX 78299 USA

**Keywords:** Mammary stem/progenitor cell, Enrichment, Digestion method, Surface marker, Flow cytometric analysis

## Abstract

Murine mammary stem/progenitor cell isolation has been routinely used in many laboratories, yet direct comparison among different methods is lacking. In this study, we compared two frequently used digestion methods and three sets of frequently used surface markers for their efficiency in enriching mammary stem and progenitor cells in two commonly used mouse strains, C57BL/6J and FVB. Our findings revealed that the slow overnight digestion method using gentle collagenase/hyaluronidase could be easily adopted and yielded reliable and consistent results in different batches of animals. In contrast, the different fast digestion protocols, as described in published studies, yielded high percent of non-epithelial cells with very few basal epithelial cells liberated in our hands. The three sets of markers tested in our hands reveal rather equally efficiency in separating luminal and basal cells if same fluorochrome conjugations were used. However, the tendency of non-epithelial cell inclusion in the basal cell gate was highest in samples profiled by CD24/CD29 and lowest in samples profiled by CD49f/EpCAM, this is especially true in mammary cells isolated from C57BL/6J mice. This finding will have significant implication when sorted basal cells are used for subsequent gene expression analysis.

## Background

Murine mammary stem/progenitor cell isolation has been routinely used in many laboratories since the first two reports of mammary stem cell isolation in 2006 (Shackleton et al. 2006; Stingl et al. 2006). Although three leading laboratories working in this field have summarized their methods, highlighted their differences and similarities and also discussed the reasoning behind the approaches they have taken in a recent review (Smalley et al. 2012), direct comparison among these different methods is lacking. Investigators new to this field often need to compare and try out these different methods and select one that works in their laboratories. We, as a relatively new group to this field, had encountered many difficulties in establishing this protocol when we first started this work, particularly in choosing a digestion method and surface markers. In addition, when we submitted papers for publication, we have had reviewers questioning us why we had used one set of markers (e.g., CD24/CD49f) instead of another set of surface markers (e.g., CD24/CD29) for enriching mammary stem and progenitor cells. To address some of these shared obstacles by new investigators in this field, we compared two frequently used digestion methods and three sets of frequently used surface markers for their efficiency in enriching mammary stem and progenitor cells in two commonly used mouse strains, C57BL/6J and FVB. Mammary tissues were obtained from these mice according to established guidelines approved by the Institutional Animal Care and Use Committee at the University of Texas Health Science Center.

## Findings

### Mammary tissue digestion methods: slow versus fast

**Fig. 1:**
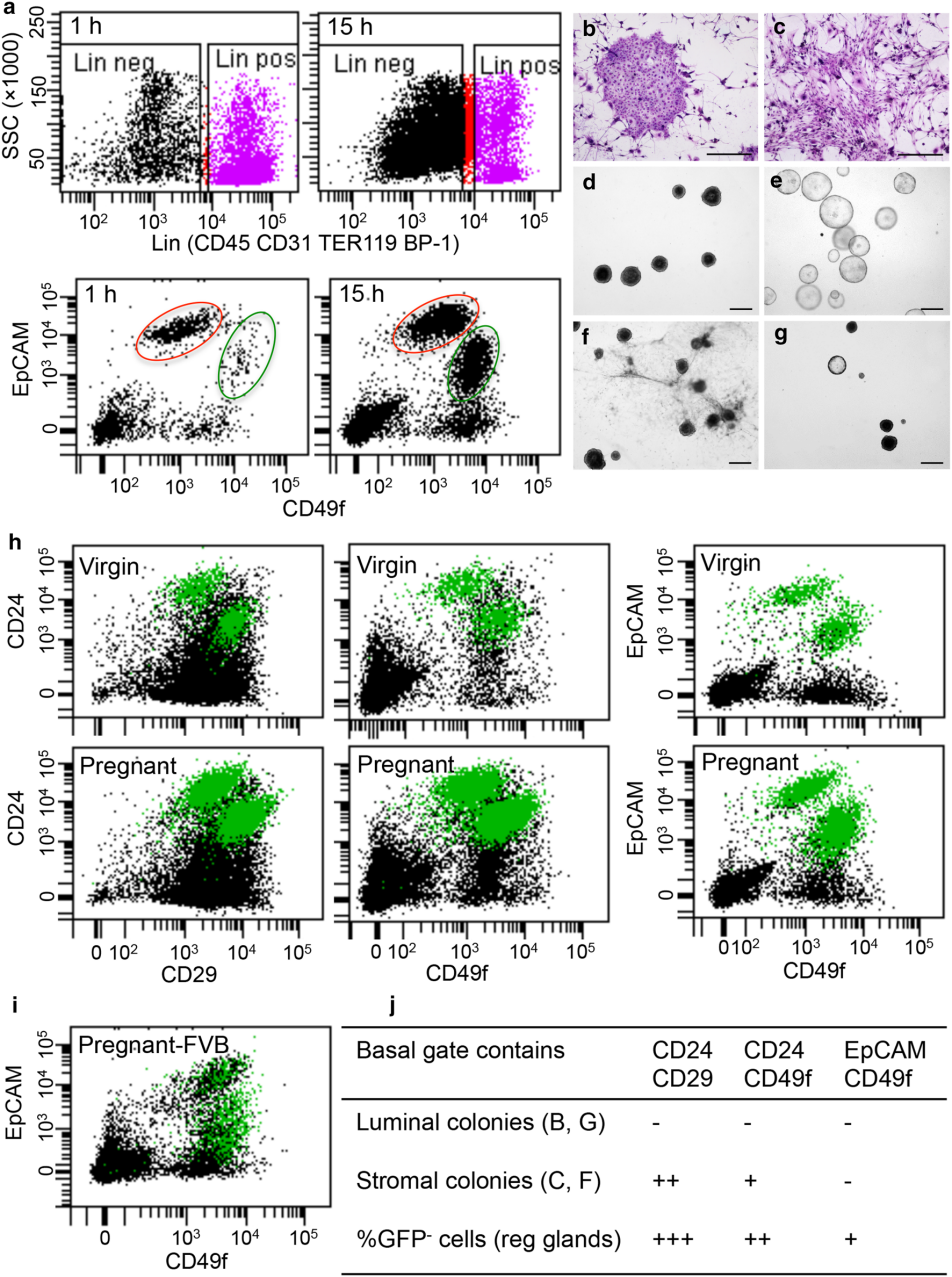
Effects of digestion method and surface marker on the purity of stem/progenitor cell isolation. **a** Mammary glands from two mice were divided into two equal portions with one thoracic gland and one inguinal gland from one mouse mixed with another mouse, and used for fast (1 h) and slow (15 h) digestion protocols. The experiment was repeated three times with a total of 6 C57BL/6J mice. Flow cytometry profiles showing predominance of non-epithelial lineage (CD45, CD31, TER119, BP-1) positive cells (Lin pos) in tissues digested with fast protocol versus the predominance of Lin^−^ (Lin neg) cells in tissues from the same mice digested with slow protocol. Within the Lin^−^ cells, basal (*green circles*) and luminal (*red circles*) cells are characterized by EpCAM^+^CD49f^hi^ and EpCAM^hi^CD49f^lo^, respectively. Fast digestion yielded much lower percentage of basal cells than slow digestion. **b** Representative epithelial colony formed by luminal progenitor and **c** non-epithelial colony formed by stromal cells on the irradiated NIH3T3 fibroblast feeder layer (2D assay). **d** Solid organoids formed by basal-like stem cells, and **e** hollow organoids formed by luminal progenitors after they were seeded in Matrigel (3D assay). **f** Branching-like structures formed by non-epithelial stromal cells in the 3D assay. **g** Inclusion of luminal progenitor in basal fraction showing hollow organoid formation in 3D assay. **h** Flow cytometry profiles showing Lin^−^ mammary cells isolated from regenerated glands derived from GFP transgenic C57BL/6J donor mice. Thus, epithelial cells were GFP^+^ (*green dots*) and niche stromal cells were GFP^−^ (*black dots*). Regenerated glands of virgin mice were pooled from ten transplants, and regenerated glands of pregnant mice (day 16) were pooled from four transplants. Isolated single cell suspension from these transplants were divided into three equal portions for flow profiling comparison using CD24/CD29, CD24/CD49f, and EpCAM/CD49f. **i** Flow cytometry profile showing the use of EpCAM/CD49f in regenerated pregnant glands derived from GFP FVB donor mice. **j** A summary table showing basal cell gate profiled by the three pairs of surface markers (CD24/CD29, CD24/CD49f, EpCAM/CD49f) contains a different number of non-epithelial stromal cells evaluated by the 2D and 3D in vitro assays as well as the GFP-marked regenerated glands (reg glands). *Scale bars* in **b–g** 500 μm

Protocols of mammary tissue dissociation vary a lot among different laboratories including gland processing such as with or without mincing or minced with scissors or tissue chopper, digestion medium and enzyme, and digestion time and method. In general, tissue digestion was carried out either rapidly in a short incubation time of 45 min to 2.5 h (Shackleton et al. 2006; Guo et al. 2012; Huo and Macara 2014; Joshi et al. 2010; Milani et al. 2013; Sleeman et al. 2006) or more slowly in a longer incubation time between 5 and 15 h (Stingl et al. 2006; Prater et al. 2013; Tao et al. 2011; Zeng and Nusse 2010). We initially tested out various fast digestion methods and found that the mammary tissue cannot be completely digested if glands were not minced or chopped into a homogenous paste and/or without constant agitation, resulting in very low cell yield. When we improved the tissue mechanical dissociation by extending chopping time and increased agitation during the digestion process, we did obtain high cell yield (e.g., 3–33 million cells from two thoracic and two inguinal glands of a mouse). However, a very small fraction of cells (≤10 %) were lineage-depleted (Lin^−^) epithelial cells (Fig. 1a). The Lin^−^ was defined as cells depleted of endothelial (CD31) and hematopoietic (CD45 and TER119) cells. Furthermore, the percentage of basal cells characterized with EpCAM^+^CD49f^hi^, the fraction highly enriched with mammary stem cells, was significantly lower with the fast digestion method (0.5 ± 0.1 % of total cells, Mean ± SE, n = 3) when compared with the slow digestion protocol (8.0 ± 0.8 %, *P* = 0.016, paired *t* test) using mammary tissues from the same mice (Fig. 1a).

We found the slow digestion protocol (overnight digestion for 15 h) using the gentle collagenase/hyaluronidase (Cat. # 07919, Stemcell Technologies) can be reliably used to isolate adequate epithelial cells (yielding 50–80 % Lin^−^ cells) from different strains of mice regardless of age (young or old) or body composition (lean or obese). With this protocol, tissue mincing and sample agitation are not necessary, thus it is also easier for new investigators to follow the procedure and generate consistent results. In contrast, most fast digestion protocols involve tissue mincing and sample agitation, and without knowing all the details, we found those protocols more difficult in obtaining consistent results and in reproducing similar cell quantity and quality as reported by investigators who initially developed these protocols.

### Stem/progenitor cell enrichment: surface marker comparison

For stem/progenitor cell enrichment, three sets of surface markers are currently used by different groups, namely the combination of CD24/CD29 (Shackleton et al. 2006), CD24/CD49f (Stingl et al. 2006), and EpCAM/CD49f (Prater et al. 2013). We used single cell suspension isolated from glands of the same mice for direct comparison of stem/progenitor cell enrichment with these paired markers. To exclude possible interference by fluorochromes, we used the same fluorochrome conjugation of allophycocyanin (APC) for CD24 (M1/69, rat) and EpCAM (G8.8, rat), the same conjugation of R-phycoerythrin (PE) for CD49f (GoH3, rat) and CD29 (HMβ1-1, Armenian hamster), and the streptavidin conjugated Pacific Blue (6,8-difluoro-7-hydroxycoumarin fluorophore, PB) for biotinylated CD31/CD45/Ter119/BP1 antibody cocktail (tagging Lin^+^ cells). The criteria used to differentiate the effectiveness of different paired markers are the purity of sorted basal and luminal cells and quantity of stem/progenitor cells from same amount of total mammary cells isolated from same mice. The purity can be assayed by in vitro colony formation on the irradiated NIH3T3 fibroblast feeder layer (2D assay) where luminal progenitors form epithelial colonies (Fig. 1b) and non-epithelial stromal cells form elongated mesenchymal-like colonies (Fig. 1c) or by the sphere formation and differentiation assay we developed recently (Dong et al. 2013), which involves imbedding of spheres formed in suspension culture in the BD Matrigel (3D assay) where basal-like stem cells formed solid organoids (Fig. 1d), luminal progenitors formed hollow organoids (Fig. 1e), and non-epithelial stromal cells formed branching-like structures (Fig. 1f). If sorted basal or luminal cells contained stromal cells, we would see non-epithelial 2D (Fig. 1c) or 3D colonies (Fig. 1f). If sorted basal cells contained luminal cells or vice versa, we would see hollow organoids formed by luminal progenitors among solid organoids derived from basal-like stem cells or vice versa (Fig. 1g). In general, basal-like stem cells do not form epithelial colonies in the 2D assay, thus presence of large numbers of 2D colonies in basal cells would indicate luminal cell inclusion in the basal cell gate.

By examining the basal and luminal cells sorted with the three sets of paired markers in cells obtained from the FVB mice, we found similar numbers of basal-like stem cells and luminal progenitors both from the virgin or pregnant glands (data not shown). For purity, all these three pairs yield high purity of luminal cells that are free of basal or stromal cells as we did not see non-epithelial colonies in the 2D assay and solid organoids in the 3D assay. However, for the basal cells, the highest inclusion of non-epithelial cells was found in samples sorted using CD24/CD29, followed by CD24/CD49f and EpCAM/CD49f, especially in pregnant glands, which may in part be due to not so clear separation of basal cells from the stromal cells (see below).

### Surface marker comparison using GFP-labeled regenerated glands

An alternative way of testing the efficiency of these three pairs of markers in purifying epithelial basal and luminal cells from the primary mammary cells is to use cells isolated from the regenerated glands derived from transplanted stem cells of transgenic green fluorescent protein (GFP) C57BL/6 mice (thus all epithelial cells are GFP^+^). Our findings revealed that essentially all GFP^+^ cells congregate in Lin^−^CD24^+^EpCAM^+^CD29^+^CD49f^+^ compartment, validating that epithelial cells were enriched efficiently with all these three pairs of surface markers. However, we also observed a significant portion of GFP^−^ cells (non-epithelial cells) within cells gated in the luminal or basal fractions (Fig. 1h). In general, we found higher portion of GFP^−^ cells within the basal gate than the luminal gate. The GFP^−^ cells within the luminal gate did not form any in vitro colonies in the 2D or 3D assay, indicating that these cells are neither colony forming stromal cells nor colony forming epithelial cells with GFP expression silenced. Within the basal cell gates, we found the inclusion of highest percentage of GFP^−^ cells in samples profiled by CD24/CD29, followed by CD24/CD49f, and the inclusion of least GFP^−^ cells in samples profiled by EpCAM/CD49f. These GFP^−^ cells did form in vitro stromal colonies in the 2D and 3D assays, indicating inclusion of stromal cells. The highest inclusion of GFP^−^ cells in samples profiled by CD24/CD29 may mainly be due to high CD29 expression in the stromal cells. Of note, although we found that EpCAM/CD49f yielded the highest purity of basal and luminal cells from both virgin and pregnant glands in C57BL/6J mice, its ability in segregating basal cells from stromal cells in mammary glands from pregnant FVB mice appears poor (Fig. 1i).

## Conclusions

To conclude, in our hands, the slow overnight digestion method using gentle collagenase/hyaluronidase could be easily adopted and yielded reliable and consistent results in different batches of animals. In contrast, the different fast digestion protocols, as described in published studies, yielded high percent of Lin^+^ cells with very few basal cells liberated in our hands. Of note, we did not mean to say that the fast digestion method would not work; rather it is more difficult to obtain desirable results without knowing every bit of details from the original authors. The three sets of markers tested in our hands reveal rather equally efficiency in separating luminal and basal cells if same fluorochrome conjugations were used. However, the tendency of non-epithelial cell inclusion in the basal cell gate was highest in samples profiled by CD24/CD29 and lowest in samples profiled by CD49f/EpCAM (Fig. 1j), this is especially true in mammary cells isolated from C57BL/6J mice. This finding will have significant implication when sorted basal cells are used for subsequent gene expression analysis.

## Slow digestion protocol for mammary stem cell isolation

1. Sacrifice mice under anesthesia and spray outside of mouse with alcohol (e.g., isopropanol solution) before making incisions.
2. Dissect the tissue in a sterile hood and use a pair of separate sterile scissors to remove mammary glands.
3. Transfer dissected mammary glands (usually from one mouse) to a 50-mL Falcon tube containing 5 mL digestion medium (one part 10X gentle Collagenase/Hyaluronidase [Cat#07919] mixture with nine parts of complete EpiCult^®^-B Medium (Mouse) supplemented with 5 % FBS [Cat#06100] and 0.05 mg/mL gentamycin).
4. Loosen the tube cap, and incubate in a 37 °C, 5 % CO_2_ incubator overnight (15 h) without any vortex.
5. Close the Falcon tube cap, brief vortex for 15 s, add 10 mL cold HF (HBSS [Cat#37250] + 2 %FBS), and spin for 5 min (0.4 rcf at 4 °C). First remove fat with cutted tip, and then dump supernatant.
6. Lyse red blood cells: resuspend cell pellet in 2 mL cold HF with regular pipettor tip for well mix, add 8 mL ammonium chloride (Cat#07850), mix well, sit on ice for 5 min, spin for 5 min (0.4 rcf at 4 °C), and dump supernatant.
7. Add 2 mL of pre-warmed trypsin–EDTA (Cat#07901) to the pellet such that the organoids are well suspended. Gently pipette continuously with a 1000-μL tip for 2-3 min. Add 10 mL cold HF, spin for 5 min (0.4 rcf at 4 °C), and discard supernatant.
8. Resuspend cell pellet in 2 mL of pre-warmed (37 °C) dispase (Cat#07913) and add one tenth of the volume of 1 mg/mL DNase I (200 Ul) (Cat#07900). The sample is then triturated for 1–3 min using a 1000-μL pipette tip.
9. Dilute the cells with 10 mL of cold HF, filter through a 40-μm filter, spin for 5 min (0.4 rcf at 4 °C), and discard supernatant.
10. Resuspend cell pellet in 1 mL of HF or complete EpiCult^®^-B medium, and the single cell suspension is now ready for counting, antibody conjugation and flow cytometry analysis and sorting. Keep the suspension on ice for subsequent steps to prevent cells from re-aggregating.

*Note*: All reagents were purchased from StemCell Technologies unless specified otherwise. This protocol has been tested to work well in our hands for the following mouse strains: C57BL/6J, FVB, and Balb/C. Total cell yields varied with animal health condition, the number of mammary glands used per mouse, estrus cycle, age, and mouse strain. Typically we obtained on average of 4 million total mammary cells per mouse (2 thoracic glands and 2 inguinal glands) for C57BL/6J (4–6 month old, non-diestrus cycle) with a range between 2 and 7 million cells (a survey of n = 13 mice).

## Abbreviations

CD: cluster designation
EpCAM: epithelial cell adhesion molecular
Lin^−^: lineage-depleted cells
2D assay: 2 dimentional colony formation cell assay
3D assay: 3 dimentional extracellular matrix colony formation assay
GFP: green fluorescent protein
